# Highly Stable Quasi-Solid-State Sodium Batteries via Facile Grain Boundary Engineering

**DOI:** 10.1021/acsami.5c20866

**Published:** 2026-03-02

**Authors:** Baiheng Li, Peiyu Wang, Huilin Qing, Ian Baker, Weiyang Li

**Affiliations:** Thayer School of Engineering, Dartmouth College, 15 Thayer Drive, Hanover, New Hampshire 03755, United States

**Keywords:** solid-state battery, ceramic electrolyte, NASICON, sodium metal anode, long-term cycling

## Abstract

The development of ceramic solid-state electrolytes such as sodium superionic conductors (NASICON) is critical in the advancement of all-solid-state sodium batteries. However, the key fundamental issue lies in the large interfacial impedance and instability due to the mismatched mechanical properties across different battery components. Herein, we propose a novel interfacial engineering approach to tackle the challenge at the interface, where a Na^+^ conducting grain boundary complexion phase can be formed by cosintering NASICON with a thin layer of zinc oxide (ZnO) coating. The grain boundary complexion envelopes the NASICON grains and forms an ion-conducting network that enhances the ionic conductivity at the grain boundaries. Ultrastable symmetric cell cycling over 12,000 h was demonstrated, showing the efficacy in suppressing dendrite formation. Electrochemical impedance spectroscopy (EIS) measurements revealed a minimal increase in internal resistance over cycling. In addition, quasi-solid-state batteries using Na_3_V_2_(PO_4_)_3_ as the cathode, sodium metal as the anode, and cosintered NASICON as the electrolyte were assembled and tested at room temperature with no externally applied stack pressure. When cycled at 0.5 C, a high initial capacity of 116.7 mAh g^–1^ was obtained, and 93.2% capacity retention was achieved at the 1300th cycle with an average Coulombic efficiency of 99.98%. Even when cycled at 2 C, the battery maintained 95.8% of the initial capacity after 1200 cycles. Overall, this work provides insights into facile and strategic approaches to interfacial modification of solid-state batteries and shows the great potential of grain boundary engineering.

## Graphical abstract

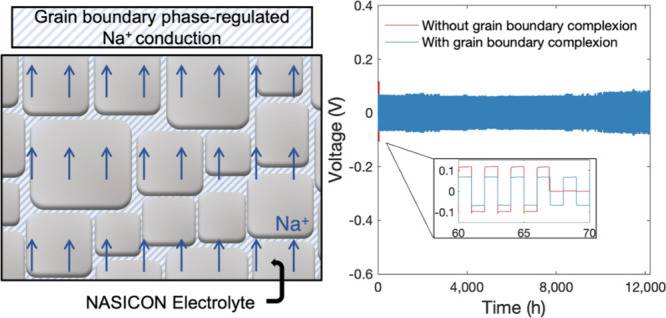

## Introduction

The consumer market for energy storage is continuously and rapidly growing globally.^1^ Technological advancements in portable electronics and electric vehicles drive up the demand in the private sector, while the urgency of energy decarbonization requires low-cost, high-capacity energy storage solutions to accommodate the discontinuous and unsteady electricity generation from renewable sources.^2^ To satisfy these needs, current lithium-ion batteries (LIBs) are facing unprecedented challenges despite the remarkable advancements that have taken place since their invention.^3^ The demand for higher power density and lower cost calls for high-capacity electrode materials and alternative chemistry. Sodium (Na) batteries are regarded as a promising candidate due to the abundance and low-cost nature of Na resources.^4^ However, potential safety risks associated with conventional LIBs, such as flammability and thermal instability, can be exacerbated in Na batteries, and these challenges need to be adequately addressed before Na batteries can be considered up to standard for mass production.^5^ To replace the organic-solvent-based liquid electrolyte that heavily contributes to these risks, a solid-state electrolyte (SE) is considered the most promising alternative due to its utmost safety features, including nonflammability and dendrite suppression. Most importantly, it allows the use of metallic Na as the anode, which has a higher capacity than any Na-hosting alloys and compounds and enables batteries with theoretical specific energies that are 3 to 4 times higher than those of commercial Li-ion batteries.^6^

Sodium-ion superionic conductor (NASICON), typically with a chemical formula of Na_1+*n*_Zr_2_Si_n_P_3–*n*_O_12_ (0 < *n* < 3), was discovered in the 1970s, which was originally developed as an electrolyte for molten Na batteries^7^ and later re-emerged to interest researchers for developing Na solid-state batteries (SSBs).^8^ Despite new material discoveries bringing new families of Na SEs such as halides and sulfides to the scene, NASICON, particularly the optimized composition Na_3_Zr_2_Si_2_PO_12_ remains a popular research focus because of its high ionic conductivity on the order of 10^–3^ S cm^–1^ as well as its superior chemical and mechanical stability.^9^ The main obstacle is poor interfacial contact and adhesion between the SE and the electrodes. The components of NASICON-based SSBs are way less malleable and reactive than the components of batteries featuring liquid electrolytes, so the formation of an SEI-like interlayer is rarely spontaneous, which is detrimental to the battery performance.^10^ Without special treatments, the electrodes and the SE tend to contact at isolated islands rather than form a continuous interface due to surface geometry. As such, the points of contact become hotspots of Na^+^ flux, leading to dendrite growth that can cause the cell to short-circuit when the localized flux eventually outpowers the SE’s ionic conductivity. Different interfacial engineering strategies have been proposed to negate this characteristic shortcoming of ceramic SEs.^11–16^ The construction of a contact-promoting artificial interlayer by thin-film deposition at the electrolyte-electrode interfaces yielded promising results in many studies.^17–20^

Recently, atomic layer deposition (ALD) has attracted extensive attention in thin film deposition because of its precise control of the film thickness in the atomic/nanometer scale.^21^ Currently, the utilization of an ALD-deposited interlayer in SSB is often limited to the deposition of simple metal oxides, and the resulting material is typically used as-is. With no further modifications following the deposition, the effects of such interlayers are limited to wettability enhancement.^22^ Despite being effective in practice, this often requires lithium or Na metal anodes to be applied to the SEs in their molten state, which not only reduces the efficiency and safety of production but can also foster the dendrite growth if voids and defects are present in the SE.^23^

Cosintering has long been the fabrication method of choice when it is desired to combine a ceramic material with a metal or multiple ceramics together as seamlessly and integrated as possible.^24^ Herein, we implemented the cosintering method to crystallize the ZnO and NASICON simultaneously and cohesively. To attain unprecedented functionalities by essentially constructing a composite ceramic SE, a nanoscale zinc oxide (ZnO) coating was applied via ALD to the NASICON SE precursor underneath. This process created a compounded precursor pellet for the cosintering process. We discovered that the cosintering process led to a grain boundary complexion composed of Na_2_ZnSiO_4_ that was distributed far deeper than the ZnO’s original thickness of a few nanometers and extended deep into the bulk. It was verified that the as-formed grain boundary complexion can mediate and redistribute localized fluxes of Na^+^, as well as impede the mechanical failure in SSBs caused by dendrites in long-term cycling. Notably, NASICON cosintered with ZnO (denoted as CS-NASICON) achieved a critical current density (CCD) of 2.6 mA cm^–2^, which is more than 3-fold than that of pristine NASICON (pristine refers to the as-prepared NASICON pellet without any further modification). Also, more than 12,000 h of stable cycling (at 0.4 mA cm^–2^ and 0.4 mAh cm^–2^) was accomplished in symmetric cells. Full cells incorporating the CS-NASICON, a Na metal anode, and a Na_3_V_2_(PO_4_)_3_ (NVP) cathode operated at room temperature (22 °C) without externally applied stack pressure exhibited excellent performance; even when cycled at 2 C, 95.8% of the cell’s initial capacity was preserved after 1200 cycles with an average CE of 99.99%. This indicates the cosintering process resulted in remarkable improvements in robustness and surface wettability in CS-NASICON thanks to the formation of the Na_2_ZnSiO_4_-rich grain boundary complexion. Overall, this work investigated the employment of the cosintering process in ceramic SE fabrication, establishing a new strategy of improving ceramic SE’s functionality by facile grain boundary engineering.

## Results and Discussion

To investigate the effect of cosintering technique, CS-NASICON was fabricated by applying ALD-ZnO coating equally to the two faces of the green-body NASICON precursor pellets before high-temperature sintering. Meanwhile, the same ALD process was also applied to sintered pristine NASICON, but with no further treatment (denoted as APS-NASICON). For both samples, ALD-ZnO layers approximately 7 nm in thickness were deposited. The fabrication processes are depicted in Figure 1. ZnO was chosen because it can be readily reduced by metallic Li or Na and then forms various phases and thus has been commonly utilized in both Li and Na battery research as a surface-modifying coating layer.^25–28^ As such, because of the overall favorable effects of ZnO coating, we hypothesized that the functionalities of ZnO coating can be further amplified by employing the cosintering technique to integrate it with NASICON, which is a commonly used technique in functional ceramic design.^24,29–31^ It is known that the metal oxide thin films deposited by ALD at a moderate temperature (100 °C) are amorphous,^25^ and hence, the synchronous crystallization with NASICON allows the newly generated grain boundary species to be both chemically and mechanically integrated with the bulk.

**Figure 1:**
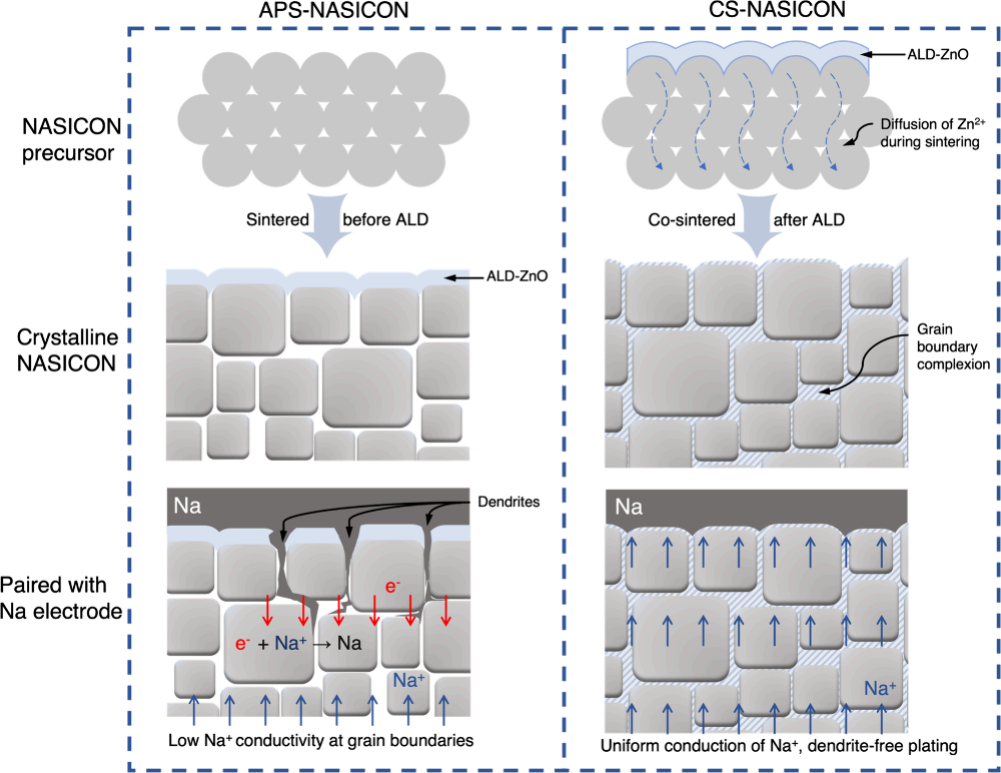
Schematics showing the fabrication processes of the modified NASICON pellets. Left: APS-NASICON, where the ALD-ZnO coating was applied post sintering, and the NASICON crystals formed independently of the ALD-ZnO layer. Right: CS-NASICON, where the nanoscale ZnO layer was absorbed into the underlying NASICON bulk after the high-temperature sintering process.

The formation of voids and pores is a common problem during the prolonged solid-phase sintering process that is required to fully crystallize ceramic SEs.^32^ This is represented by the blank space within the APS-NASICON bulk in Figure 1 and is a major cause of the reduced ionic conductivity at grain boundaries, which in turn results in localized Na^+^ fluxes and thus encourages dendrite growth. Conversely, although grain boundary complexions formed as a product of the cosintering process do not entirely eliminate the porosity, they encapsulate and interconnect individual NASICON grains in the CS-NASICON, which greatly enhances the ionic conductivity across the grain boundaries.

The X-ray diffraction (XRD) patterns of the pristine NASICON and CS-NASICON pellets are presented in Figure 2a. The most notable change due to cosintering is that the diffraction peaks of CS-NASICON shifted to lower angles. It was reported that Zn as a dopant that partially replaces Zr can greatly improve the ionic conductivity of NASICON-type electrolytes.^33–35^ However, such cases show little distinguishable difference in peak angles in doped vs undoped XRD patterns, as the change in lattice parameters resulting from doping is very small.^35^ Therefore, the observed shifting of peaks can be better explained by the strain within the sample. The initial mismatch of the thermal expansion coefficients of the two materials leads to grain growth in a constrained manner.^29^ The grain growth pattern of the bulk is hence altered despite the gradual dissolution of the ZnO layer that initially caused the strain. This is manifested as a shift of the whole XRD pattern. While the residual stress on the SE pellet’s surface was tensile as a result of the SE being “stretched” by the ZnO layer, a compressive residual stress was generated within the bulk as a response. Previously, it was discovered that residual compressive stress efficiently slows down or prevents the propagation of dendrites in SEs,^36–39^ since the creep rate of dendrites is very sensitive to stress.^37^ In SSB cycling, one of the mechanisms of dendrite propagation was found to be fissure fracture, where the tip of a dendrite exerts a massive amount of pressure on the SE due to geometric effects and eventually causes cracks that propagate far ahead of the spread of dendrites.^40^ Due to the significant difference in hardness between the Na metal and the NASICON SE, the residual compressive stress could essentially close any cracks generated by dendrites and cut off paths for this mode of crack propagation.

**Figure 2:**
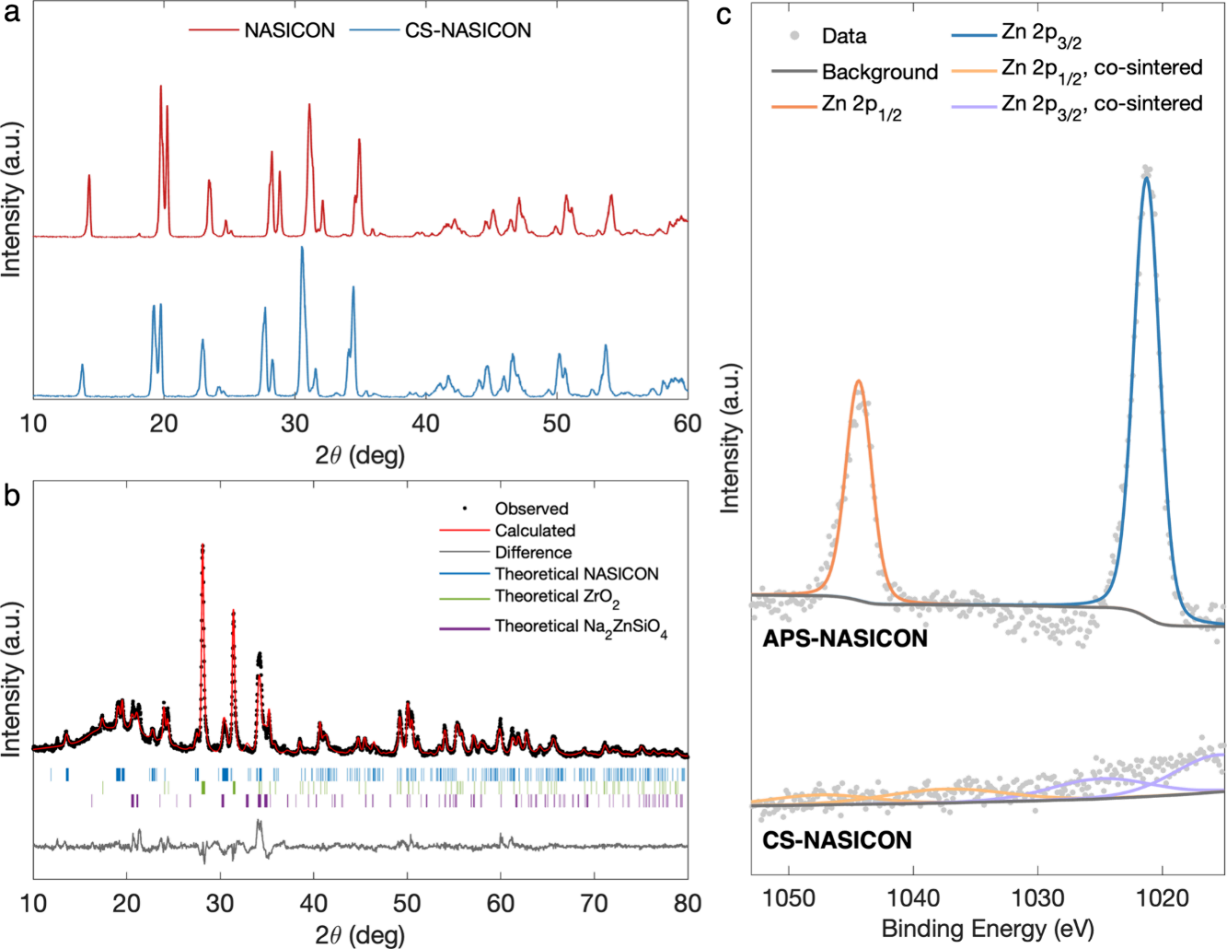
(a) XRD patterns of pristine NASICON and CS-NASICON. (b) Rietveld-refined XRD pattern of the grain boundary complexion sample shown in Figure S1e. Monoclinic NASICON (C2/c symmetry), monoclinic ZrO_2_ (P21/c symmetry), and monoclinic Na_2_ZnSiO_4_ (Pn symmetry) as phases determined by the refinement, where the small difference denotes trace phases resulting from NASICON decomposition. (c) XPS of Zn 2p in APS-NASICON and CS-NASICON at approximately 6.8 nm below the surface, plotted with a unified *y*-axis; the latter features significantly lower concentration and satellite peaks that indicate a mixed valence state due to the tetrahedral coordination of Zn^2+^ in Na_2_ZnSiO_4_.

To investigate the composition of the grain boundary complexion phase, cosintering was performed for a two-layered pellet consisting of 5 wt % ZnO powder and 95 wt % NASICON precursor. With this mass ratio, the NASICON is still considered to be the bulk phase, while the amount of ZnO is sufficient for generating the grain boundary phase in an excess amount to allow for easy characterization (Figure S1). Evidently, this phase had a far lower melting point than both NASICON (greater than 1200 °C)^41^ and ZnO (1975 °C) according to its melted and resolidified appearance. Rietveld refinement of this sample’s XRD pattern (Figure 2b) revealed that Na_2_ZnSiO_4_ was the functioning and defining species in the grain boundary complexions, while ZrO_2_ was present as the main decomposition product from the NASICON, where its weight percentage in the grain boundary complexion matches that in the NASICON. The very small difference in the observed XRD pattern and the refinement result based on the three phases (Na_2_ZnSiO_4_, ZrO_2_, and Na_3_Zr_2_Si_2_PO_12_) indicates that the grain boundary complexions contain very small amounts of other species, which indicates a good fit. The trace species represented by the difference may include phosphates as a result of NASICON’s decomposition. The ionic conductivity of sodium zinc silicate electrolytes with compositions of Na_*x*_Zn_*x*/2_Si_2‑*x*/2_O_4_ (1.25 ≤ *x* ≤ 2) was studied by Grins;^42^ when *x* = 2, the ionic conductivity was found to be 5.88 mS cm^–1^ at 327 °C. While the room temperature conductivity was not reported, this value still rivals the conductivity of many widely studied SEs for Na SSB at elevated temperatures. Additionally, it was also reported that Na_2_ZnSiO_4_ has a monoclinic crystal structure,^42^ which is the same crystal structure as NASICON at room temperature. This would promote the formation of a coherent interface between the two species. When this grain boundary complexion forms through the reaction between the nanometer-thick ZnO and the NASICON precursor substrate at 1175 °C (compared to the sintering temperature of 777 to 1052 °C reported for Na_*x*_Zn_*x*/2_Si_2‑*x*/2_O_4_),^42^ the Na_2_ZnSiO_4_ has ample time and driving force to permeate the NASICON crystal boundaries in its liquid state, resulting in a wider presence of the grain boundary complexion compared to the original nanometer range of the thickness of the ALD coating applied.

X-ray photoelectron spectroscopy (XPS) combined with depth profile analysis was used to study the variation in composition and elemental concentration at the electrolyte surface and underneath. Since the expected thickness of the as-deposited ZnO film was approximately 7 nm according to the instrumental calibration data of the ALD system, and the sputtering rate of Ar ion beam was calibrated to be 1.7 nm per min on a SiO_2_ reference substrate, 4 min of sputtering was performed, which corresponds to an observation depth of approximately 6.8 nm. The collected data were calibrated with respect to C 1s (284.8 eV). For the APS-NASICON, the narrow and sharp shapes of the Zn 2p peaks indicate that Zn is present as ZnO and is highly concentrated near the surface, while in comparison, the CS-NASICON has significantly weaker Zn 2p signals and both Zn 2p^1/2^ and 2p^3/2^ peaks broadened considerably, which may suggest the appearance of satellite peaks (Figure 2c). The widened peak shapes and the appearance of satellite peaks suggest that the Zn in CS-NASICON is no longer concentrated near the surface, and its oxidation state has changed considerably to differ fundamentally from a simple oxide. This agrees with the previous speculation that ZnO reacts with NASICON during cosintering, and the reaction product Na_2_ZnSiO_4_ filtrates down into the bulk, since the complex chemical bonding of Zn in such a case would match the XPS characteristics. Further, a strong shift in binding energy can be observed when comparing the XPS spectra of CS-NASICON and APS-NASICON (Figure S2). At a depth of 6.8 nm below the surface, the Zr 3d^5/2^ and Zr 3d^3/2^ peaks have binding energies of 186.4 and 184.0 eV in the APS-NASICON, and these values are lowered to 185.3 and 183.1 eV in the CS-NASICON, respectively (Figure S2a,e). Si 2p shifted from 103.5 to 102.8 eV (Figure S2b,f), P 2p from 139.0 and 134.4 to 133.6 and 128.7 eV (Figure S2c,g), while there is no significant change in the O 2p binding energy (Figure S2d,h). The overall chemical shift indicates that the NASICON was slightly reduced in the cosintering process, which has been proposed to promote interfacial stability in NASICON SE.^17,43^ Additionally, it was reported that a reduced NASICON has improved affinity to Na^+^, and excess Na^+^ can be accommodated by vacant Na sites in the NASICON lattice,^44^ thereby improving the ionic conductivity by increasing the size of bottlenecks for Na^+^ pathways within the NASICON crystal structure and reducing the energy barrier for the interstitial transportation of Na^+^.^45,46^

Scanning transmission electron microscopy (STEM) was used to further investigate the distribution of Zn in the grain boundary complexions formed in the CS-NASICON. A pellet was ground into fine powder using a mortar and pestle by hand, and the powdered SE pellet was sonicated in ethanol to make a dilute suspension, which was then drop-cast onto a carbon-coated copper (Cu) TEM grid. Figure 3a shows a group of particles up to a few hundred nanometers in size, in which the distributions of all elements overlap with no significant discontinuities. The absence of a pure NASICON phase (which is defined by the exclusion of Zn^2+^) in the STEM sample can be explained by the distribution pattern of the grain boundary complexions. During sample preparation, a CS-NASICON pellet was crushed into fine particles. Because the grain boundary complexion encapsulates NASICON grains, the imaged sample particles show a continuous spread of Zn^2+^ that completely overlaps with the elements (Na, Si, P, and Zr) contributed by NASICON and thus proves the hypothesized distribution of the grain boundary phase.

**Figure 3:**
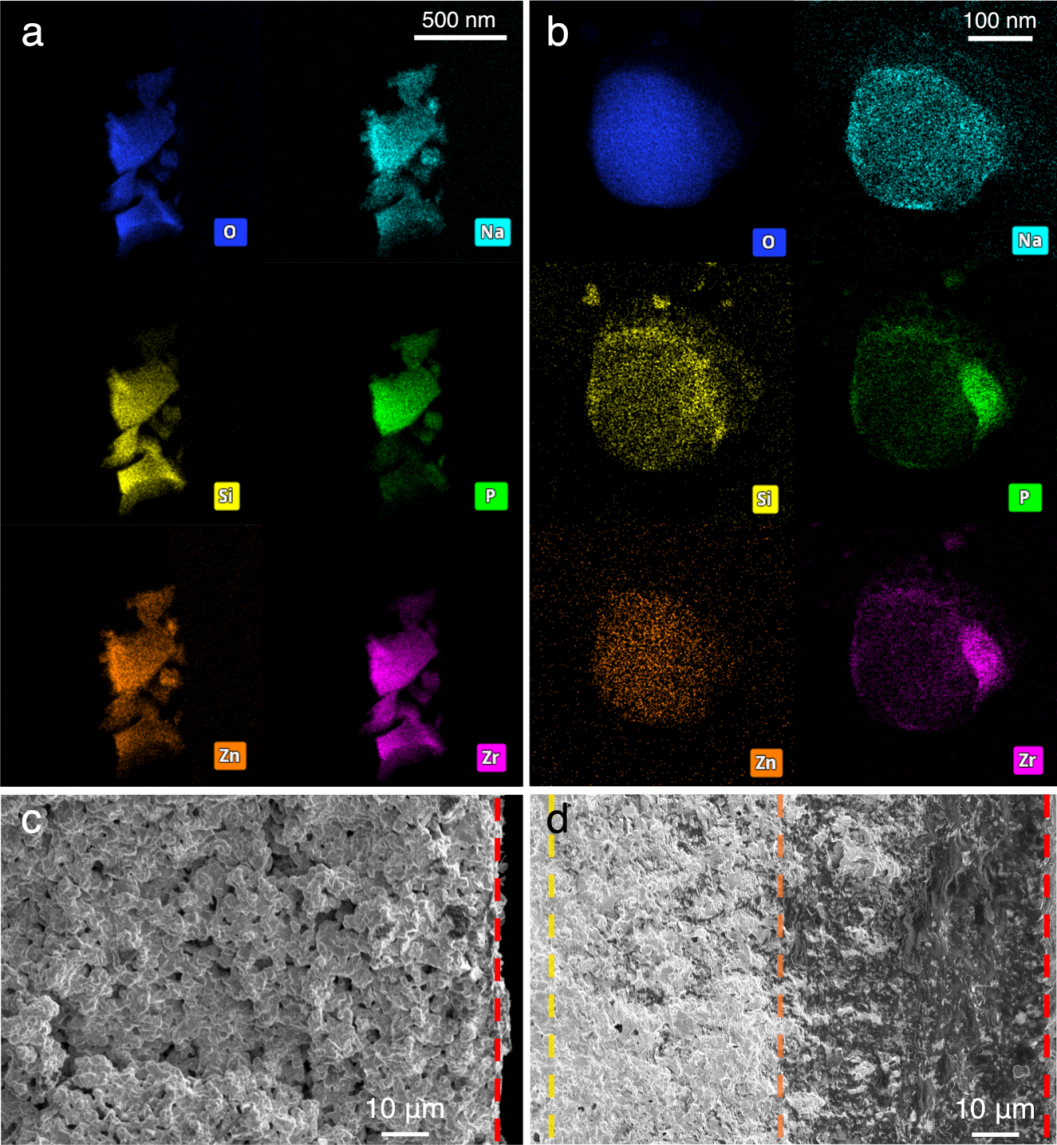
(a) STEM-EDS analysis of the distribution of O, Na, Si, P, Zn, and Zr in a collection of CS-NASICON particles. (b) Morphology of a particle resulted from low-temperature cosintering a Cu grid with preloaded NASICON precursor particles with ALD-ZnO coating. Cross-sectional SEM images of (c) pristine NASICON and (d) CS-NASICON, where the top surfaces perpendicular to the cross-section can be seen at the right side of both images (indicated by red dashed lines). The dashed orange line in (d) marks the end of the boundary layer where the grain boundary complexion phase is most concentrated (within about 50 μm beneath the surface), where the yellow dashed line marks the edge of the diffusion zone outside of which the grain boundary complexion phase could not be observed.

To demonstrate that the grain boundary complexion readily forms during the cosintering process, carbon-coated Cu TEM grids were lightly loaded with NASICON precursor, followed by an ALD-ZnO coating, and were annealed at 950 °C. Although the annealing temperature was limited by the melting point of Cu and was not sufficient for either of the species to fully crystallize, the resulting crystalline growth of NASICON and grain boundary complexion can still be distinguished (Figure 3b). As P and Zr are not a part of the predicted composition of the grain boundary complexions (the signals of P and Zr are contributed by NASICON), the overlapping distributions of O, Na, Si, and Zn in the particle indicate that a new phase of Na_2_ZnSiO_4_ distinct from NASICON has formed after this low-temperature cosintering process. Additionally, even though the ALD-ZnO coating layer initially follows the contours of the substrate, after cosintering, the Zn had a discontinuous spread and migrated to where Zr and P were absent. These observations eliminate the possibility that cosintering would result in the doping of NASICON, which would require all elements to overlap. Scanning electron microscopy (SEM) was employed to survey the surface characteristics of SEs fabricated with different procedures (Figure S3). All samples appeared dense with occasional submicron-sized superficial voids, meaning that the crystallization behavior of NASICON was not largely impacted in the control groups. Additionally, the cross sections of both pristine NASICON and CS-NASICON were also imaged with SEM to study the distribution pattern of the grain boundary complexion (Figure 3c,d). Cross sections were created by breaking NASICON SE pellets apart mechanically, and in both images, the edges of the top surfaces can be seen at the right side. In the pristine NASICON, the cross-section appeared to be similar to the top surface. In comparison, the grain boundary complexion phase in the CS-NASICON can be seen as a distinct, darker color with a glassy appearance. The contact between the grain boundary complexion and NASICON phases is very intimate near the top surface, which eliminates all voids just below the surface. The grain boundary complexion is most concentrated within 50 μm beneath the surface (Figure 3d) but can reach as deep as around 100 μm from the surface.

To evaluate the long-term stability of the Na plating and stripping of each sample, Na//Na symmetric cells were assembled and cycled at 0.4 mA cm^–2^ with a capacity of 0.4 mAh cm^–2^. Testing was performed at room temperature, and no externally applied stack pressure was applied. This moderate cycling current was carefully chosen to compare the performance of each sample during long-term cycling. Pristine NASICON was able to cycle for approximately 67 h before a short circuit occurred (Figure 4a), while the APS-NASICON maintained cycling for more than 700 h (Figure S4). While these two samples both cycled with an overpotential of approximately 0.12 V, the ZnO coating was observed to greatly lengthen the cycle life of the APS-NASICON. Additionally, to study the potential effect of ZnO’s crystallinity, a heat treatment was applied to APS-NASICON to crystallize the originally amorphous ZnO coating at a temperature lower than the original sintering process (900 °C) to prevent any chemical changes in the bulk phase. This sample, which is correspondingly denoted as HT-APS-NASICON, was designed as a direct comparison to the APS-NASICON sample, where the main difference is the crystallinity of the ZnO coating. The HT-APS-NASICON was cycled under the same conditions in symmetric cells. Even though the overpotential of the symmetric cell was reduced to about 0.08 V (Figure S5), the interface became glaringly unstable, as the cell quickly short-circuited after 50 h of cycling. In contrast, over 12,000 h of stable Na plating and stripping was achieved with the symmetric cell with the CS-NASICON, and the voltage plateaus of 0.08 V remained smooth over the course of time, which indicates that the interfacial chemistry is highly reversible with little Na dendrite formation (Figure 4a). Electrochemical impedance spectroscopy (EIS) measurement was performed after the battery was cycled for 2,800 cycles (corresponding to 5,600 h) as well as after 5,000 cycles (10,000 h) (Figure 4b), and a negligible difference was observed when compared to the measurement of the same cell prior to the start of cycling. This observation verifies that the superb cycle life of the cell was not “soft shorts” in disguise.^47^

**Figure 4:**
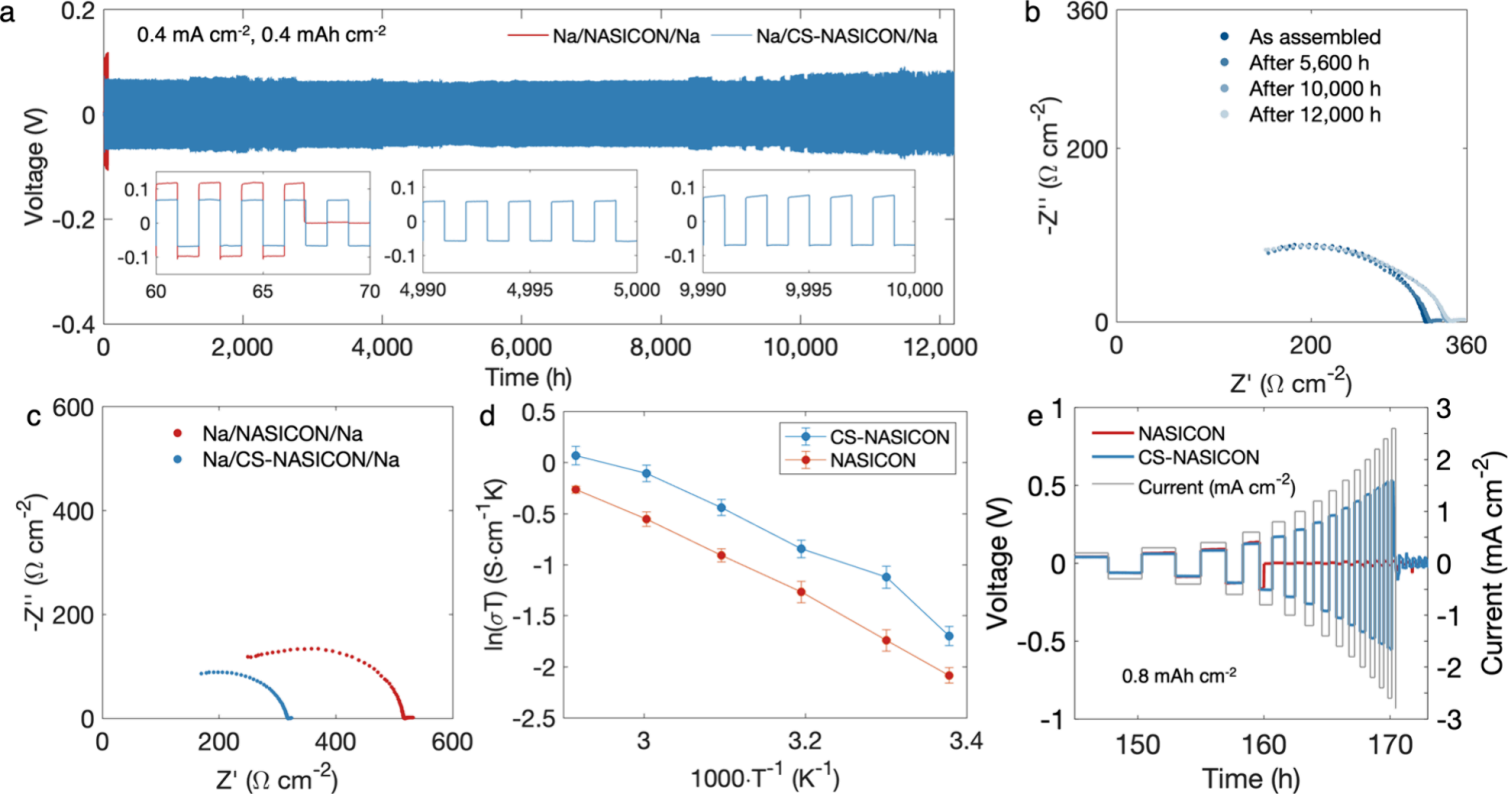
(a) Na/NASICON/Na and Na/CS-NASICON/Na symmetric cells cycled at 0.4 mA cm^–2^, 0.4 mAh cm^–2^. Insets show zoomed-in voltage curves at various cycles. (b) EIS of Na/CS-NASICON/Na as assembled, after 5,600 h of cycling (2,800 cycles), after 10,000 h of cycling (5,000 cycles), and after 12,000 h of cycling (6,000 cycles). (c) EIS of Na/NASICON/Na vs Na/CS-NASICON/Na symmetric cells right after cell assembly. (d) Arrhenius plot (ln­(σT) vs T^–1^) of pristine NASICON and CS-NASICON, where σ represents the total ionic conductivity. (e) Critical current densities of Na/NASICON/Na (0.8 mA cm^–2^) and Na/CS-NASICON/Na (2.6 mA cm^–2^) measured at a controlled capacity of 0.8 mAh cm^–2^. All electrochemical testing was performed at 22 °C.

The interfacial impedances of the pristine NASICON, the CS-NASICON, the APS-NASICON, and the HT-APS-NASICON were compared in Na//Na cells using EIS. The results are shown in Figures 4c and S6, where it is evident that the cosintering reduces the interfacial impedance. In EIS spectra, three resistance components are expected to be observed as individual semicircles theoretically: bulk resistance (*R*_bulk_), grain boundary resistance (*R*_gb_), and interfacial resistance (*R*_int_). Here, in the EIS spectra tested with Na//Na symmetric cells, the high-frequency *R*_bulk_ was not captured due to instrumental limitations. In the low-frequency region, only one arc was observed, which is due to the overlapping time constants of *R*_gb_ and *R*_int_. When they overlap, the individual semicircles representing each resistance element combine into an elongated arc.^48^ Although difficult to resolve, the existence of both resistance elements can be verified by the aspect ratios of the arcs that are greater than 2 (Figure S7) compared to a semicircle’s aspect ratio (width/height) of 2.^49^ The total resistance values (*R*_total_ = *R*_bulk_ + *R*_gb_ + *R*_int_), which are characterized by the *x*-intercepts of the arcs, were used to compute values of the conductivities (σ) for the Arrhenius plots (ln­(σT) vs T^–1^) in Figure 4d. The activation energies of pristine NASICON and CS-NASICON were found to be 0.312 and 0.292 eV, respectively.

It was reported that a layer of ZnO coating on the SE surface can lead to enhanced ionic conductivity solely because of improved surface wettability.^20^ Even though crystalline ZnO has better wettability than amorphous ZnO (Figure S8), no significant reduction in resistance was observed when comparing the HT-APS-NASICON to the APS-NASICON, where the crystallinity of the surface ZnO increased after the prolonged heat treatment. Figure S9 compares the difference in surface wettability between the pristine NASICON, the APS-NASICON, and the CS-NASICON when in contact with molten Na. The images were taken 10 s after the initial contact. Both the pristine NASICON and the APS-NASICON display similar contact angles, suggesting that the as-deposited amorphous ALD-ZnO does not modify the surface’s affinity to Na, despite the quick reaction between Na and the ZnO layer that was indicated by the formation of a brown compound. In contrast, the Na droplet on the CS-NASICON surface has a much smaller contact angle. Considering that the heat treatment results in a more significant reduction in contact angle than cosintering (Figure S8) but did not result in a comparable improvement electrochemically (Figures S4 vs S5), it can be determined that the mechanism that improves CS-NASICON’s cycle stability is not solely dependent on wettability.

CCD measurement was performed for all samples in the Na//Na symmetric cells using a controlled capacity of 0.8 mAh cm^–2^ with the current ramping up starting from 0.02 mA cm^–2^ (Figures 4e and S10). The CCD of the CS-NASICON was determined to be 2.6 mA cm^–2^, which is more than triple that of the pristine NASICON’s CCD value of 0.8 mA cm^–2^. The CCDs of APS-NASICON and HT-APS-NASICON were 1.4 and 0.15 mA cm^–2^, respectively. When comparing the CCD values of the APS-NASICON and the CS-NASICON to those of the pristine NASICONs, it is clear that applying ZnO coating alone is already quite constructive for improved performance, which aligns with many previous studies.^19,20^ Here, by simply changing the order of fabrication steps, a much better result can be achieved by the cosintering process. Up until the failure of the Na/NASICON/Na symmetric cell, its overpotential closely matched that of the Na/CS-NASICON/Na cell, which indicates that the improved performance of the latter did not come from any fundamental changes in the ionic conductivity of the bulk phase but rather from the improved cohesivity in the grain boundaries. CCD testing using a fixed duration of 15 min per half cycle was also conducted for pristine NASICON and CS-NASICON (Figure S11a). When the cells used to conduct this test were disassembled, CS-NASICON’s ability to suppress dendrites was visualized: compared to pristine NASICON (Figure S11b), which shows severe lateral growth of dendrites at 0.8 mA cm^–2^ and a darkened Na anode, and CS-NASICON (Figure S11c) shows a lesser degree of dendrite propagation at 1.4 mA cm^–2^.

To better understand the synergistic effect of the grain boundary complexion on the NASICON SE’s electrochemical properties, NVP was chosen as the cathode material and metallic Na as the anode to assemble full cells. Because neither externally applied stack pressure^50–52^ nor elevated testing temperature was a part of the testing condition, a trace amount (5 μL cm^–2^) of wetting agent (1 M NaPF_6_ in diglyme) was applied to the cathode laminate prior to battery assembly. The cells were tested between 2.4 and 3.6 V. Impressively long cycle life was also observed for NVP/CS-NASICON/Na. When cycled for 1300 cycles at 0.5 C, the cell maintained a specific capacity that is 93.2% of the initial value, and an average Coulombic efficiency (CE) of 99.98% was achieved (Figure 5a). As a comparison, the specific capacity of the NVP/NASICON/Na faded rapidly before its failure at 420 cycles. The charge–discharge voltage profiles of these cells at different cycle numbers are shown in Figure 5b.

**Figure 5:**
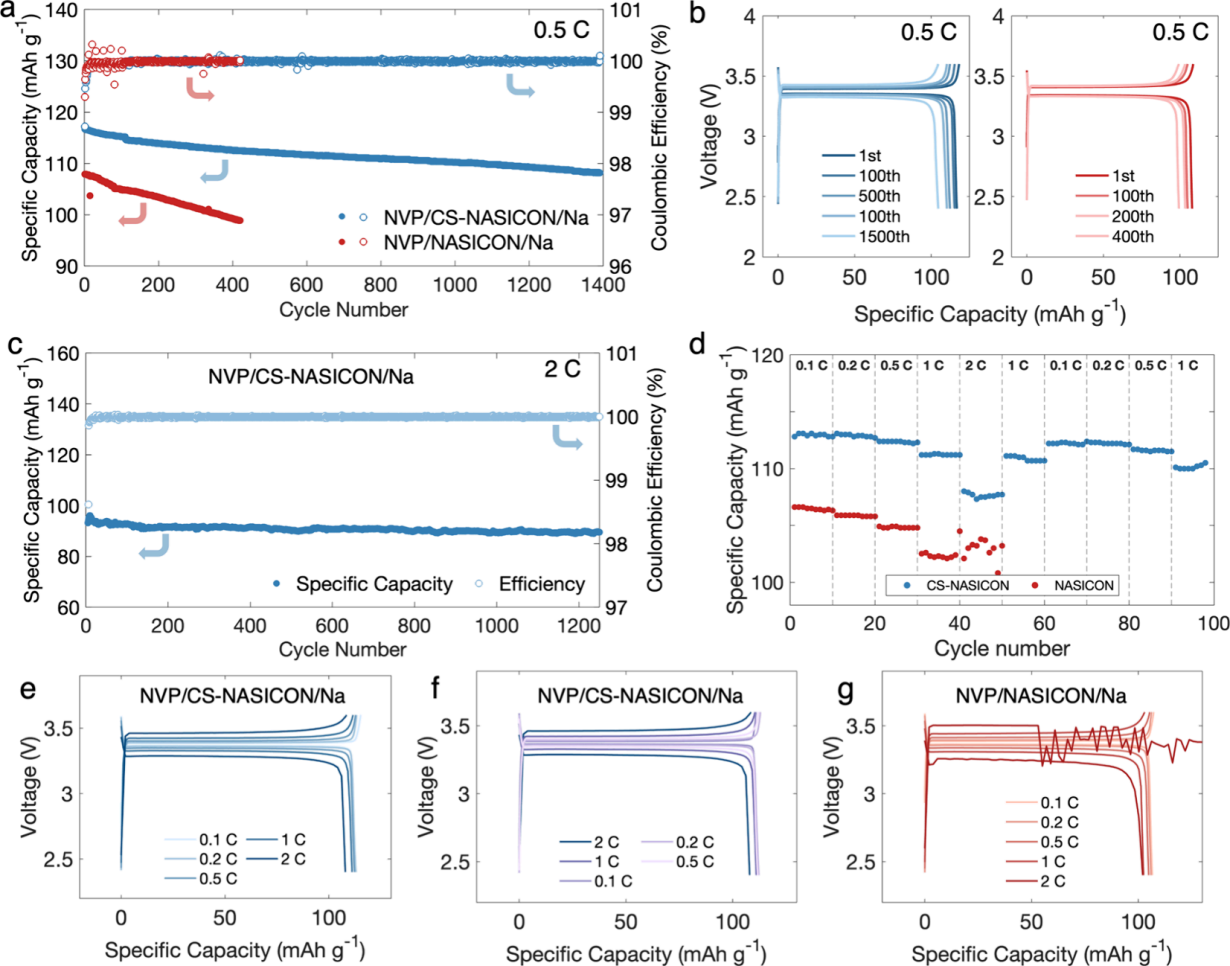
(a) Comparison of electrochemical cycling performance of NVP/CS-NASICON/Na and NVP/NASICON/Na using a charging rate of 0.5 C with no externally applied stack pressure. (b) Charge–discharge curves of samples in (a) as functions of cycle number. (c) Electrochemical cycling performance of NVP/CS-NASICON/Na cycled at 2 C with no externally applied stack pressure. (d) Rate performance of NVP/CS-NASICON/Na vs NVP/NASICON/Na under various charging rates with no externally applied stack pressure. (e, f) Charge–discharge curves of NVP/CS-NASICON/Na in (d) as a function of charging rate. (g) Charge–discharge curves of NVP/NASICON/Na in (d) as a function of charging rate. All batteries were tested at 22 °C. Active material loading of the NVP cathodes was approximately 1.6 mg cm^–2^.

Higher charging rates of 1 and 2 C were also tested for the NVP/CS-NASICON/Na (Figures S12 and 5c). After 1200 cycles at 2 C, the cell was able to preserve 95.8% of its initial specific capacity (89.3 vs 93.2 mAh g^–1^ initially) with an average CE of 99.99%, which overshadows the performance at 1 C, where the cell was able to preserve only 85.2% of its initial specific capacity (79 vs 93.8 mAh g^–1^ initially) after 1200 cycles. The charge–discharge curves of the cell cycled at 1 and 2 C are displayed in Figures S13 and S14, respectively. The greater ability to retain capacity even at a high rate of 2 C indicates that the interfacial chemistry is highly stabilized by the grain boundary complexions, and little side reactions occur at the interface even when Na^+^ transport remained rapid for thousands of hours, which is evident from the minimal capacity fade and high CE. Here, CS-NASICON demonstrates great potential for being utilized in battery applications where rapid delivery of power is required.

Additionally, the rate performance of the full cells was evaluated at charging rates of 0.1 0.2, 0.5, 1, and 2 C, and in this testing sequence the NVP/CS-NASICON/Na cell exhibited specific capacities of 113, 112.9, 112.4, 111.2, and 107.7 mAh g^–1^, respectively, which greatly surpasses the capacities of the Na/NASICON/Na cell tested in the same manner (Figure 5d) and were very close to NVP’s theoretical capacity of 118 mAh g^–1^. It is notable that the capacity of NVP/CS-NASICON/Na recovered to 110.9 and 112.2 mAh g^–1^, respectively, when the sequential testing was complete, and the charging rate was reverted to 1 and then 0.1 C, demonstrating excellent reversibility. The charge–discharge profiles of the NVP/CS-NASICON/Na cell are shown in Figure 5e, where the charging rate increases, and Figure 5f, where the charging rate decreases. Figure 5g shows the charge–discharge profiles of the NVP/NASICON/Na cell, where the failure of the cell at 2 C can be observed. Additionally, the changes in EIS spectra of NVP/NASICON/Na and NVP/CS-NASICON/Na cells were compared over the course of 100 cycles at 0.5 C (Figure S15). The internal resistance of the NVP/NASICON/Na cell steadily increased, which indicates the deterioration of interfacial stability, while the NVP/CS-NASICON/Na cell was able to maintain a relatively high ionic conductivity due to its stabilized ion conduction.

## Conclusions

This work investigated the cosintering technique and its application in ceramic electrolyte fabrication. Grain boundary complexions, a product of the cosintered NASICON and the nanoscale ALD-ZnO coating, infiltrated the bulk of the SE pellet and enveloped the NASICON grains. Despite its low weight percentage compared to NASICON, it acts like a glue to greatly strengthen the grain boundaries and prevent fissure fracture of the SE induced by Na^+^ dendrite growth. Characterization of the grain boundary complexions revealed the primary component to be Na_2_ZnSiO_4_, which was discovered in the 1970s as a sodium SE. The cosintering strategy proved to be greatly beneficial to the NASICON SE design since it alleviates dendrite formation and maintains a cohesive interface between the electrodes and the SE over thousands of hours of high-rate cycling. As a result, the cycle life of Na SSB was significantly lengthened, and superb capacity retention was observed at a very competitive charging rate (95.8% capacity retention at 2 C after 1200 cycles). When cycled at a 0.5 C, the battery with CS-NASICON had a high initial specific capacity of 116.7 mAh g^–1^, which is rarely seen in SSB research. Cosintering has shown its potential in ceramic materials fabrication;^29–31^ however, within the realm of battery research, so far it has only been attempted as a strategy to combine cathode materials with the SE in all-solid-state batteries (ASSB) with less than satisfactory results due to uncontrollable side reactions at elevated temperature.^53^ Grain boundary engineering is an important method in refining the electrochemical and structural properties of solid-state electrolytes for achieving highly stable interfaces and resistance to dendrite formation.^54^ Our findings showcase cosintering as a facile and highly effective approach of grain boundary engineering and provide new insights for overcoming challenges at the interfaces of ASSBs.

## Materials and Methods

### NASICON Electrolyte Synthesis and Preparation

The synthesis of the solid-state electrolyte Na_3_Zr_2_Si_2_PO_12_ started with mixing a stoichiometric ratio of Na_2_CO_3_ (Sigma-Aldrich, ≥99.5%), ZrO_2_ (Sigma-Aldrich, 99%), SiO_2_ (Sigma-Aldrich, ∼99%), and NH_4_H_2_PO_4_ (Sigma-Aldrich, ≥98.5%) in a high-energy ball mill (SPEX SamplePrep 8000 M Mixer). A ball-to-powder ratio of 4:1 was used, with an equal mass of ethanol added to the mixture to increase the yield. The mixture was milled for 2 h with breaks between each 40 min of running time to prevent overheating of the sample. The resulting powder was then collected, dried at 70 °C overnight, and subsequently calcined at 950 °C for 8 h in a tube furnace in open air. Afterward, the calcined sample was ball milled for 2 h with 0.5 wt % polyethylene glycol (Alfar Aesar, MW = 20,000) as a process control agent. The powder was then pressed into pellets that weighed 0.5 g each and were 12.7 mm in diameter by using an applied uniaxial pressure of 24 MPa. The CS-NASICON was fabricated by applying a ZnO coating on NASICON precursor pellets using an ALD system (ANRIC technologies, AT-410) using N_2_ as the carrier gas, diethyl zinc (DEZ) as the precursor, and water as the oxidant. Each ALD cycle included 3 pulses of DEZ and 2 pulses of water, followed by purging of the chamber. The thickness of the ALD coating was calibrated by measuring the actual thickness of metal oxide deposited on silicon wafers with an ellipsometer (Gaertner Scientific L104b). The resulting pellets were then sintered at 1175 °C for 10 h. The APS-NASICON was fabricated by depositing ZnO on sintered pristine NASICON pellets with 70 ALD cycles on each side. The HT-APS-NASICON was fabricated by annealing APS-NASICON for 10 h at 950 °C. After sintering, the average thickness and diameter of the pristine NASICON were 1.38 and 12.27 mm, respectively; for the CS-NASICON, its thickness and diameter were 1.32 and 12.33 mm, respectively.

### Material Characterization

The crystal structures of the NASICON and NASICON-CS were characterized using a Rigaku 007 X-ray diffractometer with the diffraction patterns collected between 10° and 60° with a scan rate of 2.0°/min with Cu Kα radiation (0.1541 nm). The surface morphologies were analyzed using an FEI Helios scanning electron microscope operated at 2 kV. Particle morphology analysis and energy-dispersive X-ray spectroscopy (EDS) were carried out using a Thermo Fisher Talos scanning transmission electron microscope in STEM mode. XPS and depth profiling were performed by using a PHI Versaprobe II scanning XPS microscope. Depth profiling was performed on a 3 × 3 mm^2^ area with an etching rate of 1.7 nm per min for 10 min and then 10.8 nm per min for an additional 75 min. The Rietveld refinement was conducted using JADE software with MDI-500 and COD-2024 databases.

The effect of ZnO’s crystallinity on wettability was demonstrated using ALD-ZnO (approximately 7 nm) deposited on Si wafer. The high crystallinity sample was fabricated by applying a heat treatment (950 °C, 8 h) to the as-deposited amorphous ALD-ZnO sample. Then, the change in wettability with respect to crystallinity was shown using water droplets of equal volumes for the two samples. The contact angles between molten Na and pristine NASICON, APS-NASICON, and CS-NASICON SEs were compared by dropping molten Na onto the SEs’ surfaces at 180 °C in an argon-filled glovebox.

### Electrochemical Measurements

To measure the ionic conductivity of the NASICON and CS-NASICON electrolyte pellets, about 3 nm of gold was sputtered onto both sides of the pellets by using a sputtering system (Hummer 6.2), and the pellets were then sandwiched between two stainless steel discs for measurement by using an electrochemical workstation (VMP3, Bio-Logic Science Instruments). Symmetric Na//Na cells in the typical 2032-type coin cell configuration were assembled after preheating the electrolyte pellet and the coin cell components at 90 °C for 40 min. NVP purchased from MSE Supplies LLC was used as the cathode for full cell testing. NVP was mixed with carbon black and PVDF in a 7:2:1 mass ratio, and the loading of the active material was approximately 1.6 mg cm^–2^. Na/NVP cells were assembled in a similar fashion as the symmetric cells but with the addition of 5 μL of liquid electrolyte of 1 M NaPF_6_ in diethylene glycol dimethyl ether (diglyme, Sigma-Aldrich). All coin cell testing was performed at room temperature (22 °C) using a battery tester (CT3002A, Wuhan LAND Electronics Co., LTD). All electrochemical measurements were conducted at room temperature (22 °C) unless otherwise noted.

## Supplementary Material



## Notes

Digital images of NASICON samples; phase composition analysis of grain boundary complexion; XPS spectra of Zr, Si, P, O; SEM images of NASICON samples; additional electrochemical characterizations of APS-NASICON and HT-NASICON samples; surface wettability comparison of as-deposited ALD-ZnO coating and heat-treated ALD-ZnO coating; additional full cell cycling data; and EIS data after various cycles (PDF)
