# Supplementary material for: Highly Stable Quasi-Solid-State Sodium Batteries via Facile Grain Boundary Engineering
Source: ACS Appl Mater Interfaces. 2026 Mar 2;18(10):14904–13. doi: 10.1021/acsami.5c20866 (PMC13006941; doi:10.1021/acsami.5c20866)
Supplement: Supplementary file 1 [file am5c20866_si_001.pdf]

Supporting Information

Highly Stable Quasi-Solid-State Sodium Batteries via Facile Grain Boundary Engineering

Baiheng Li[1], *Peiyu Wang*[1], *Huilin Qing*[1], *Ian Baker*[1], *Weiyang Li*[1]*

[1]Thayer School of Engineering, Dartmouth College, 15 Thayer Drive, Hanover, New Hampshire 03755, United States

*Corresponding author. Email: weiyang.li@dartmouth.edu

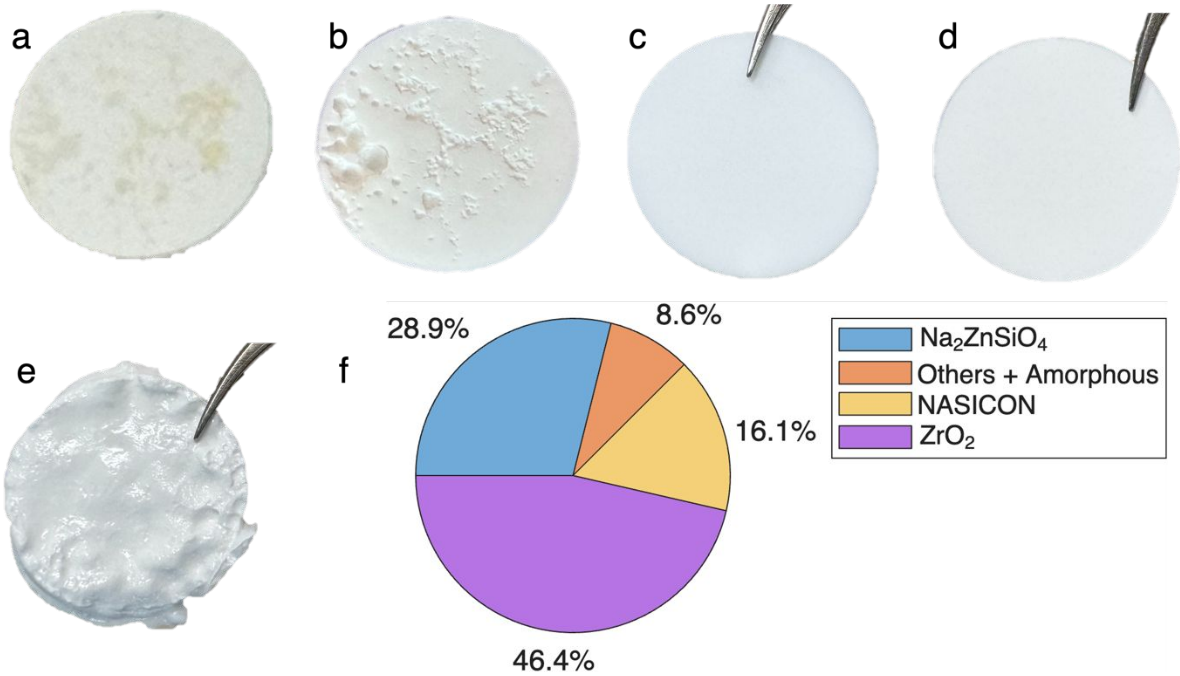

Figure S1. (a) Digital photo of the NASICON precursor pellet (0.5 g) double pressed with 5 mg ZnO unevenly sprinkled on its surface, corresponding to 1wt%, before sintering. (b) Photo of the sample shown in (a) after the co-sintering process. Note the surface indentation pattern matches the spread of ZnO in (a). (c) Photo of the sintered pristine NASICON. (d) Photo of the CS-NASICON. (e) Photo of the NASICON co-sintered with 50 mg ZnO applied to the surface of precursor pellet, corresponding to 10wt%. Dilation of the diameter is observed along with irregularity in surface morphology, and the shiny surface differs significantly from the featureless and matte surfaces of pristine and CS-NASICON, indicating the presence of a liquid phase during sintering. (f) Weight percentages calculated from Rietveld refinement results of XRD pattern of sample shown in (e). Note that the weight fraction of ZrO_2 in NASICON ($Na_3Zr_2Si_2PO_{12}$) is exactly 46.4% (molar masses of 123 g mol^{-1} and 530.55 g mol^{-1}), same as the weight fraction of ZrO_2 in this sample, indicating that a small weight fraction of ZnO additive is efficiently capable of converting NASICON to Na_2ZnSiO_4.

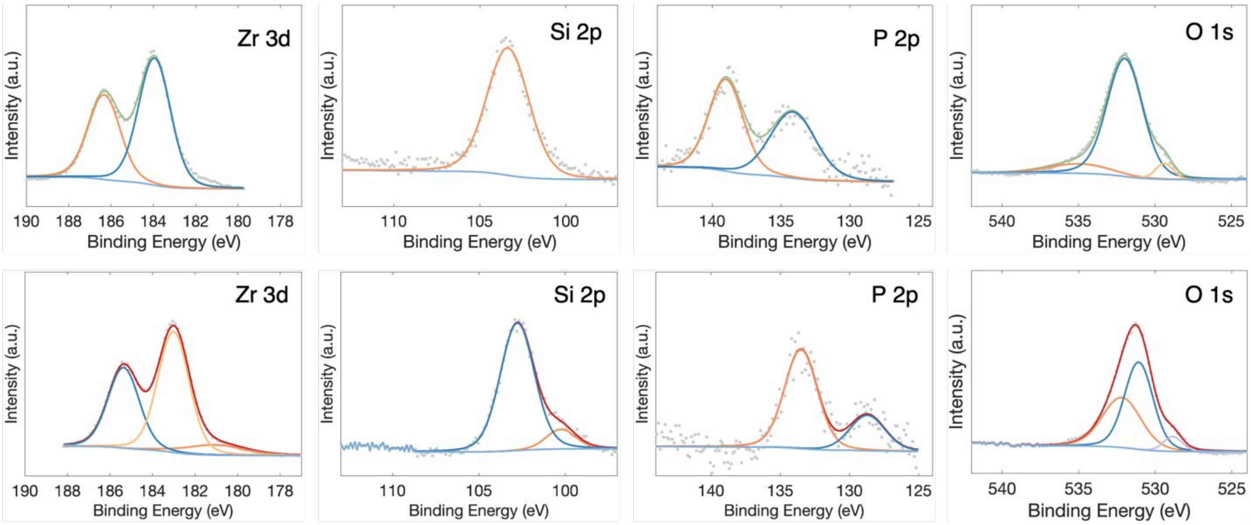

Figure S2. XPS spectra at approximately 6.8 nm below the sample surface of (a) Zr 3d, (b) Si 2p, (c) P 2p and (d) O 1s in APS-NASICON and (e) Zr 3d, (f) Si 2p, (g) P 2p and (h) O 1s in CS-NASICON, respectively.

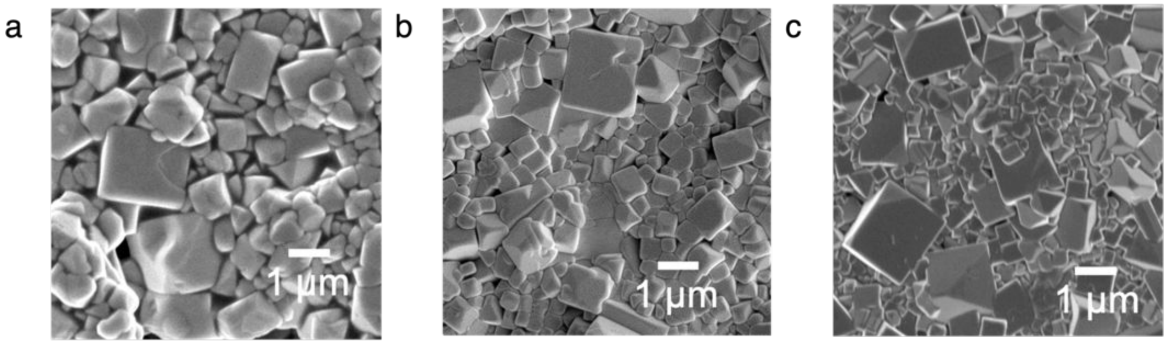

Figure S3. SEM images of (a) pristine NASICON, (b) CS-NASICON, and (c) APS-NASICON.

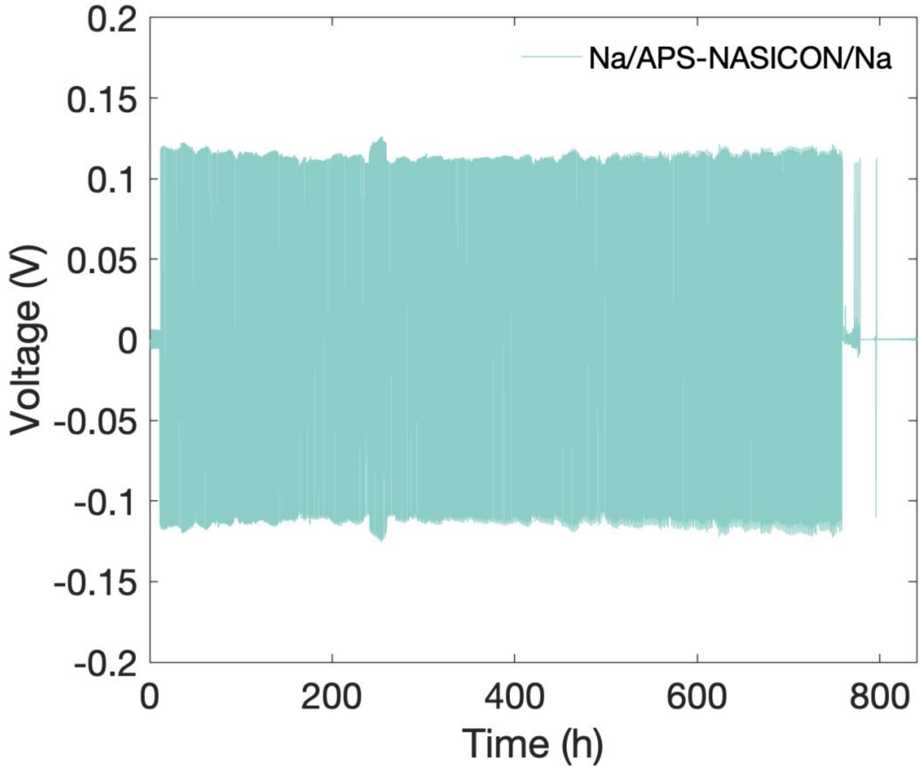

Figure S4. Symmetric cell of Na/APS-NASICON/Na cycled at 0.4 mA cm^{-2}, 0.4 mAh cm^{-2}.

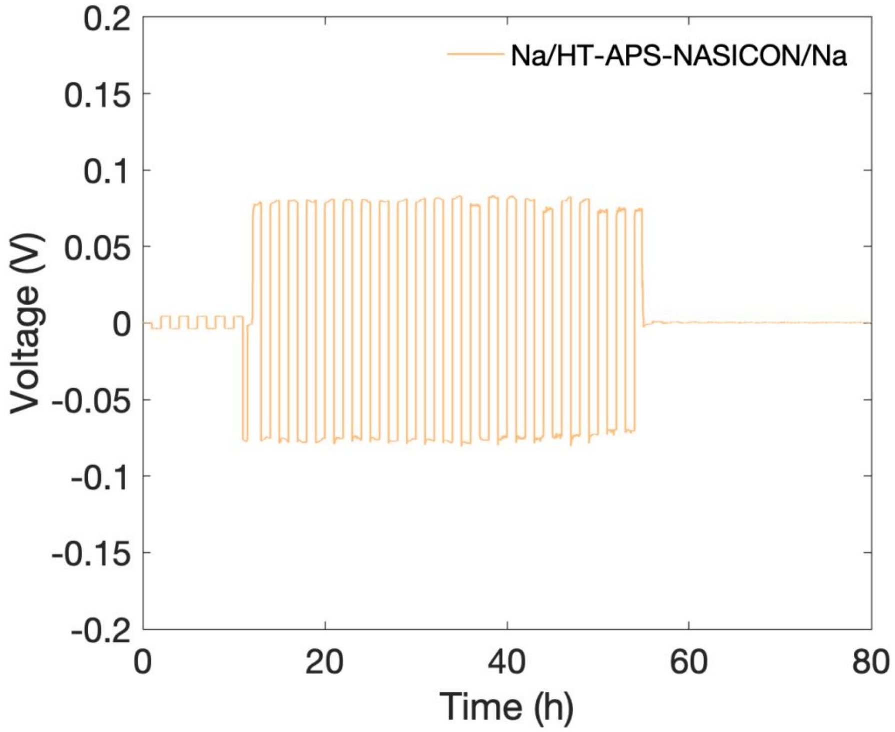

Figure S5. Symmetric cell of Na/HT-APS-NASICON/Na cycled at 0.4 mA cm^{-2}, 0.4 mAh cm^{-2}.

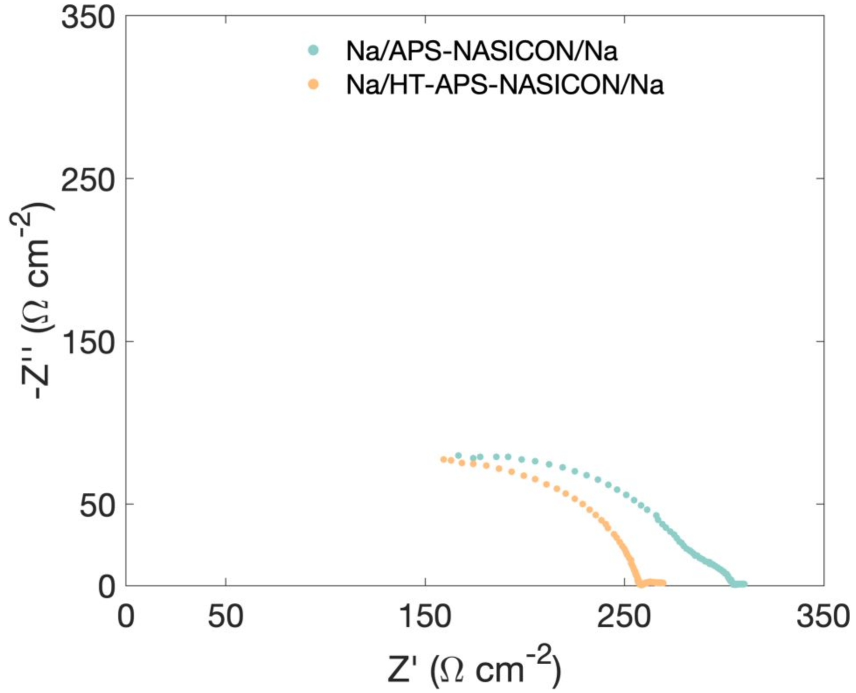

Figure S6. EIS measurements of Na/APS-NASICON/Na and Na/HT-APS-NASICON/Na symmetric cells prior to cycling.

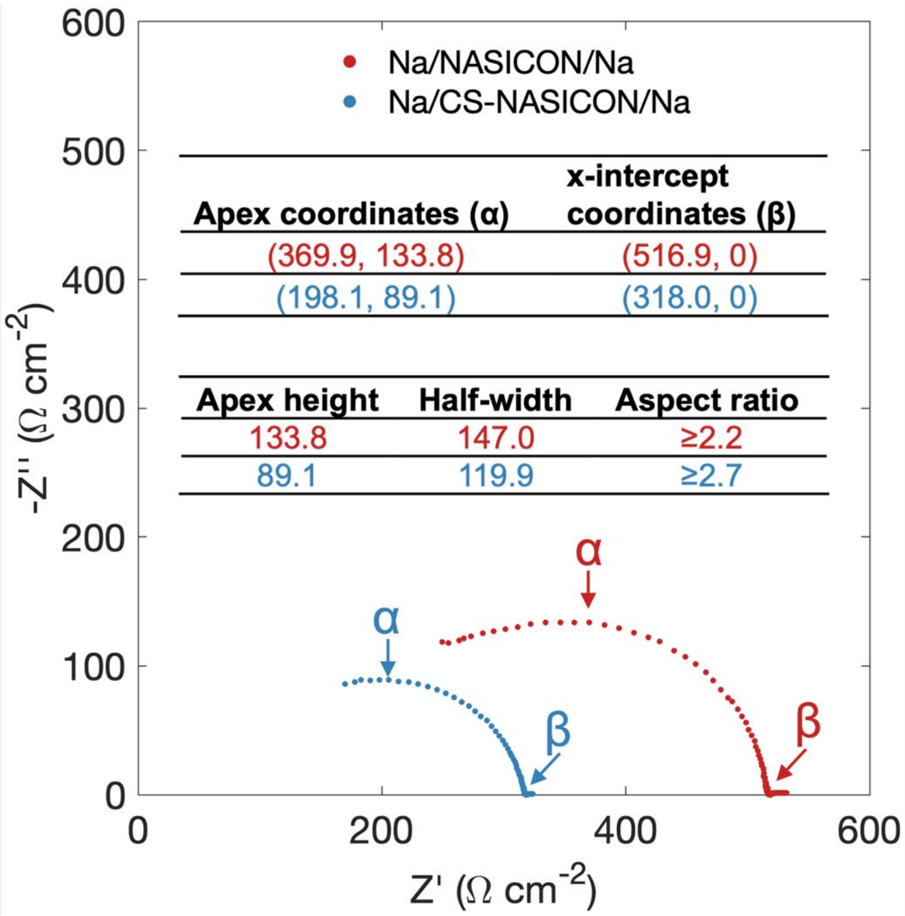

Figure S7. Graph of the resistance components in the EIS spectra of Na/NASICON/Na and Na/CS-NASICON/Na symmetric cells, where α and β indicate the apex points of arcs and x-intercepts of arcs, respectively.

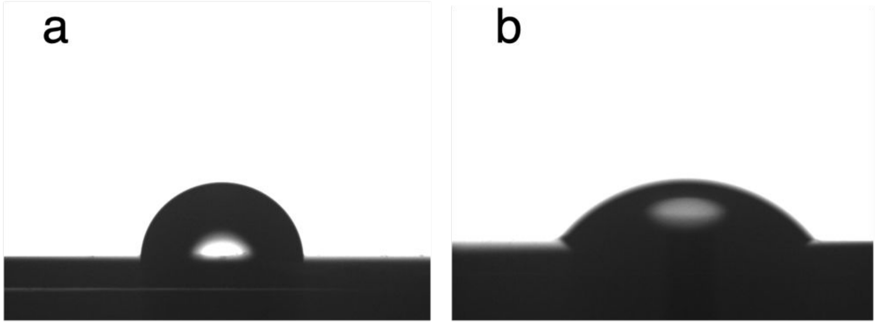

Figure S8. Photographs showing the change in surface energy with respect to the crystallinity of ZnO, demonstrated using water droplets on amorphous and crystalline ALD-ZnO thin films (approx. 7 nm) on Si wafer. (a) as-deposited ALD-ZnO (amorphous), contact angles are 84.2° (left) and 84.0° (right). (b) heat treated ALD-ZnO (crystalline), contact angles are 50.1° (left) and 54.5° (right). The decrease in contact angles and improved wettability from (a) to (b) suggests an increase in surface energy.

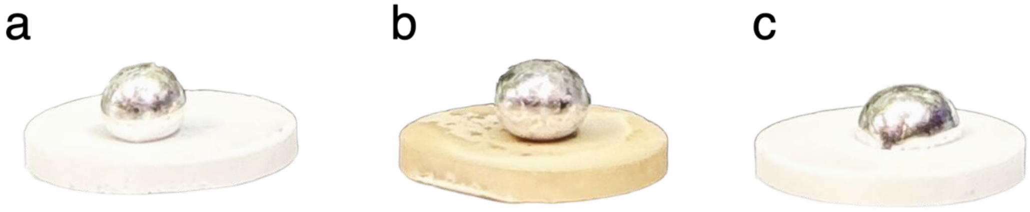

Figure S9. Optical images showing the wettability of molten sodium on (a) pristine NASICON, (b) APS-NASICON and (c) CS-NASICON.

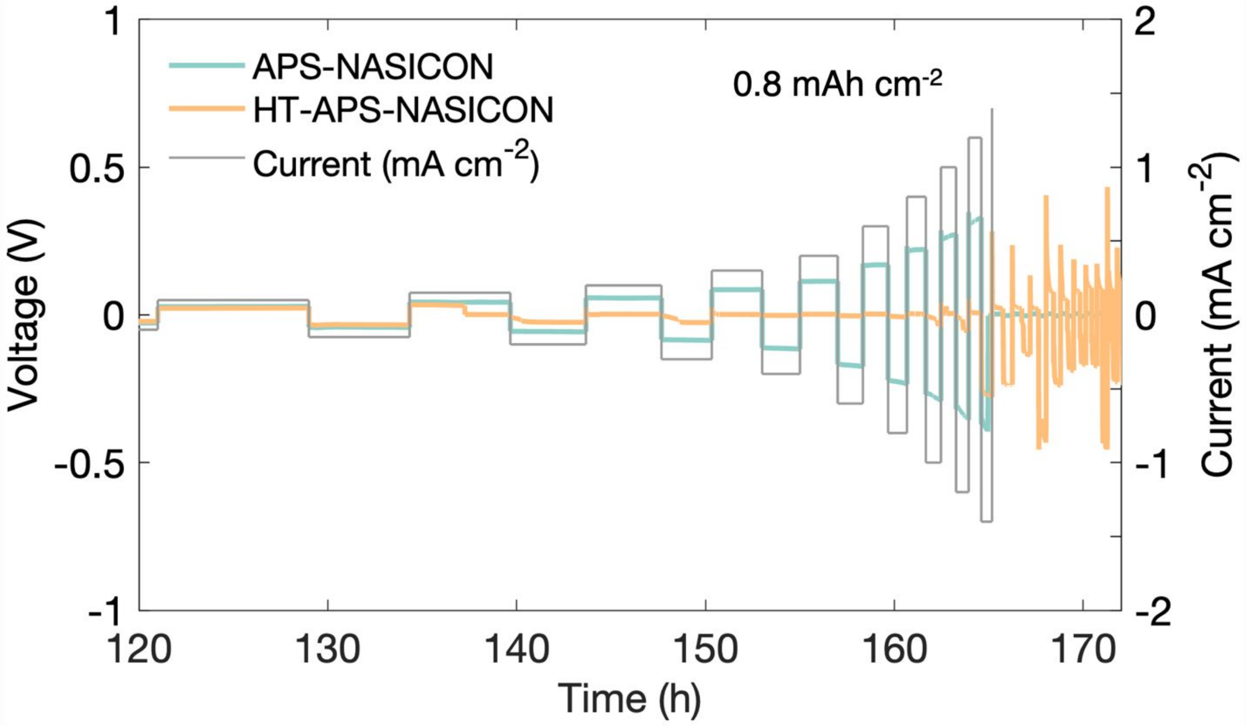

Figure S10. Critical current densities of Na/APS-NASICON/Na (1.4 mA cm^{-2}) and Na/HT-APS-NASICON/Na (0.15 mA cm^{-2}) measured at a controlled capacity of 0.8 mAh cm^{-2}.

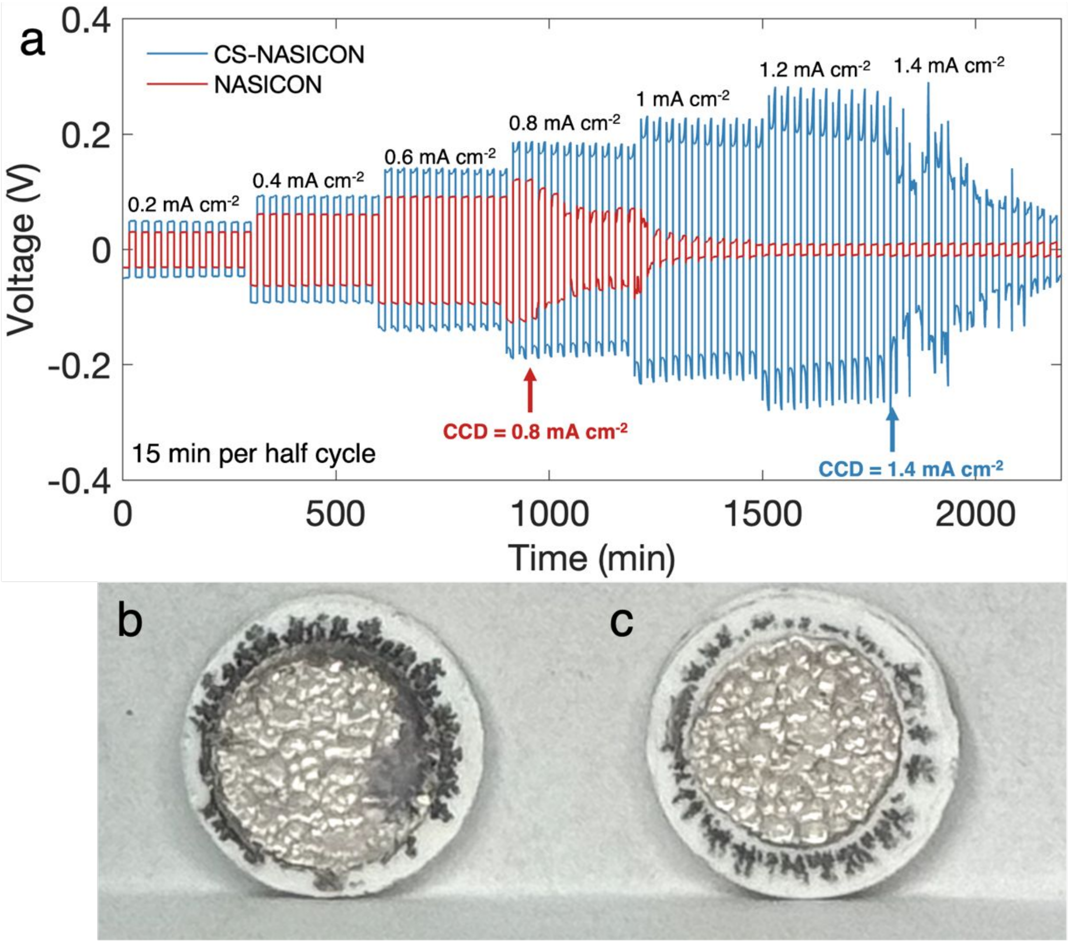

Figure S11. (a) CCD measurements of pristine and CS-NASICON conducted with a fixed charging/discharging duration of 15 min. Pristine NASICON's CCD with this method was determined to be 0.8 mA cm^{-2}, while CS-NASICON's CCD was 1.4 mA cm^{-2}. (b), (c): Post-mortem images of the pristine NASICON and CS-NASICON obtained from Na//Na cells used for CCD testing in (a).

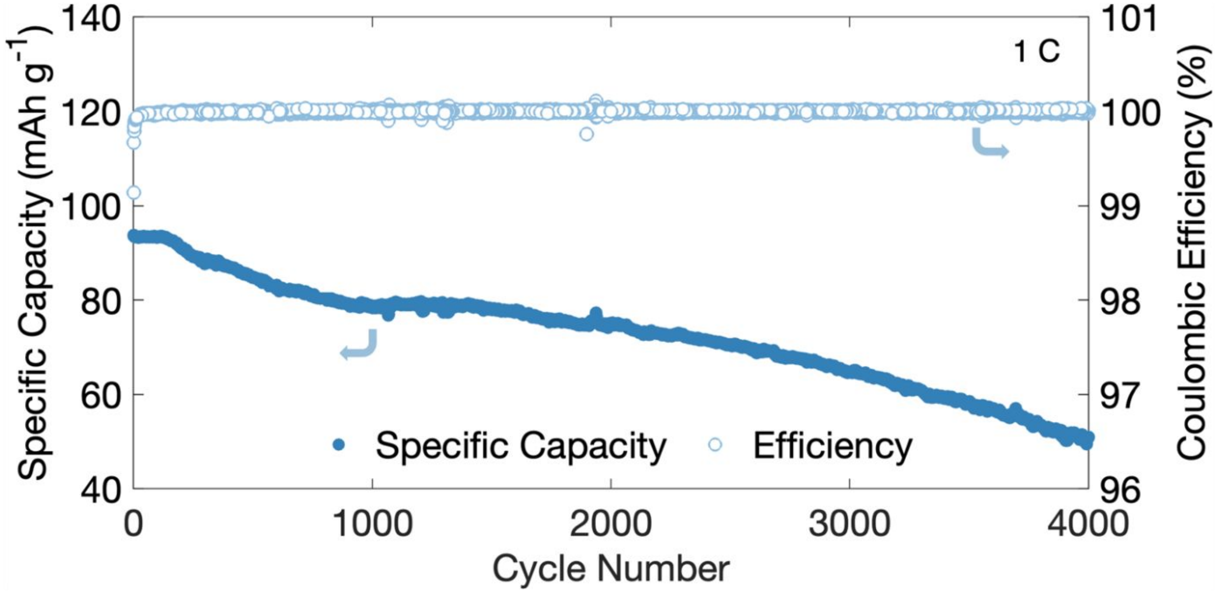

Figure S12. Electrochemical cycling performance of NVP/CS-NASICON/Na tested at 1 C at room temperature with no externally-applied stack pressure.

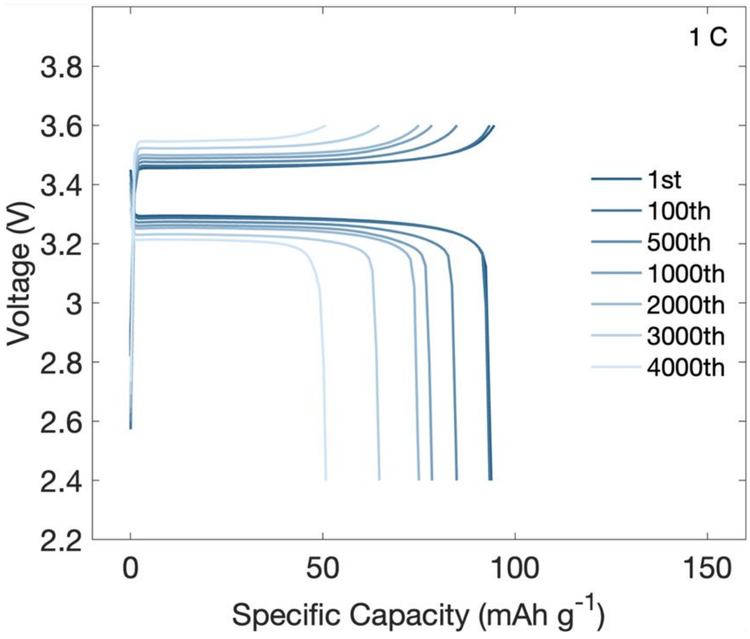

Figure S13. Charge-discharge curves of NVP/CS-NASICON/Na at different cycles, tested at 1 C at room temperature with no externally-applied stack pressure.

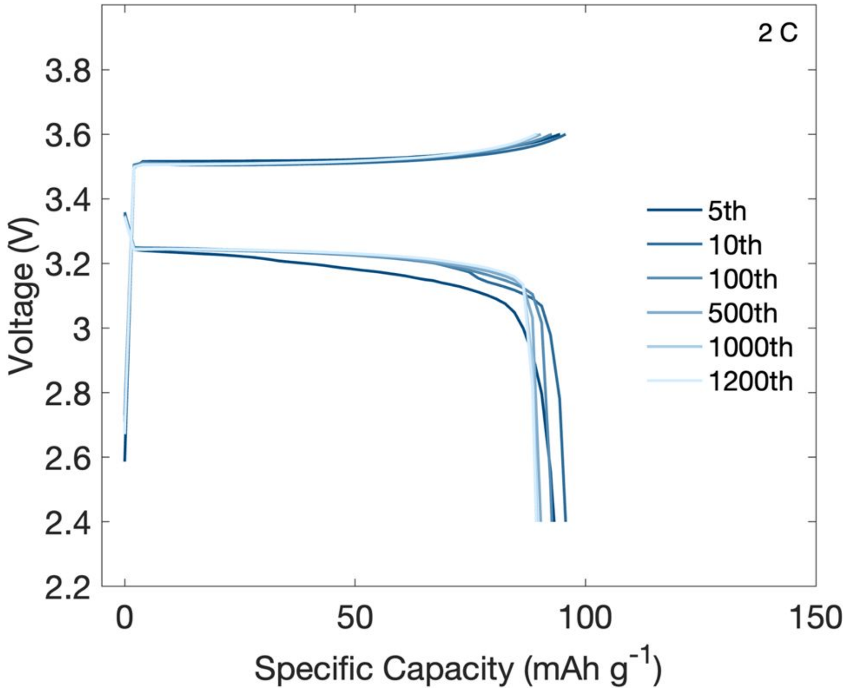

Figure S14. Charge-discharge curves of NVP/CS-NASICON/Na at different cycles, tested at 2 C at room temperature with no externally-applied stack pressure.

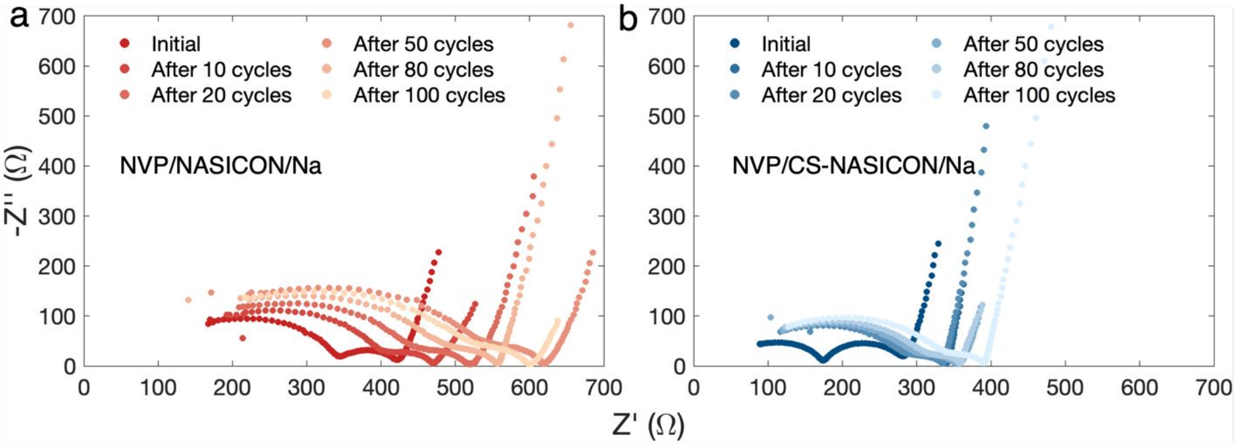

Figure S15. Evolution of internal resistance in (a) NVP/NASICON/Na and (b) NVP/CS-NASICON/Na over the first 100 cycles at 0.5 C.

Table S1. Bulk and relative densities of pristine NASICON and CS-NASICON measured using the Archimedes method, where 3.27 g cm^{-3} is the theoretical density[S1].

	Density (bulk, g cm^{-3})	Density (relative)
Pristine NASICON	2.93 ± 0.01	89.6%
CS-NASICON	3.02 ± 0.04	92.4%

References

S1. Hong, H. Y. P. Crystal structures and crystal chemistry in the system $Na_{1+x}Zr_2Si_xP_{3-x}O_{12}$. *Mater. Res. Bull.* **1976**, 11, 173–182.
